# Individual and community level factors associated with delayed first postnatal care attendance among reproductive age group women in Ethiopia

**DOI:** 10.1186/s12884-020-03523-5

**Published:** 2021-01-06

**Authors:** Achamyeleh Birhanu Teshale, Getayeneh Antehunegn Tesema, Yigizie Yeshaw, Ayenew Kassie Tesema, Adugnaw Zeleke Alem, Alemneh Mekuriaw Liyew

**Affiliations:** 1 Department of Epidemiology and Biostatistics, Institute of Public Health, College of Medicine and Health Sciences, University of Gondar, Gondar, Ethiopia; 2 Department of Physiology, School of Medicine, College of Medicine and Health Sciences, University of Gondar, Gondar, Ethiopia; 3 Department of Health Education and Behavioral Science, Institute of Public Health, College of Medicine and Health Sciences and comprehensive specialized hospital, University of Gondar, Gondar, Ethiopia

**Keywords:** Delayed first postnatal care attendance, Reproductive Age Women, Ethiopia

## Abstract

**Background:**

Postnatal care (PNC) visits provide a huge benefit for ensuring appropriate breastfeeding practices, to monitor the overall health status of the newborn, to timely diagnose and intervene birth-related complications, and to plan future family planning options. Despite delayed PNC attendance have a great impact on the survival of the mother and the newborn it still receives less emphasis. As a result, most mothers do not receive PNC services early. We, therefore, aimed to determine individual and community level factors associated with delayed first Postnatal Care attendance among reproductive age group women in Ethiopia.

**Methods:**

We used the most recent Ethiopian Demographic and Health Survey (EDHS 2016) data to determine associated factors of delayed first PNC in Ethiopia. A weighted sample of 4308 women with a live birth in the two years preceding the survey was included. A multilevel logistic regression analysis was used to analyze the data. Variables with p-value < 0.05 in the multivariable multilevel logistic regression analysis were declared significantly associated with delayed first PNC attendance.

**Results:**

In this study, both individual level and community level factors were associated with delayed PNC attendance. Among the individual level factors: having four or more antenatal care visit [Adjusted Odd Ratio (AOR) = 0.73; 95% CI: 0.59, 0.92], delivery at a health facility [AOR = 0.04; 95% CI: 0.03, 0.05], and perceiving distance from the health facility as not a big problem [AOR = 0.73; 95% CI: 0.58, 0.91] were associated with lower odds of delayed first PNC attendance. Of community level factors: being in Oromia [AOR = 2.31; 95% CI: 1.38, 3.83] and Gambela [AOR = 2.01; 95% CI: 1.13, 3.56] regions were associated higher odds of delayed first PNC attendance.

**Conclusions:**

Both individual level and community level factors were found to be associated with delayed PNC attendance. Strengthening antenatal care utilization, institutional delivery, and appropriate distributions of maternal health services in each region and areas far apart from the health facility are recommended.

## Background

To achieve the sustainable development goal three (SDG 3), that is ensuring healthy lives and promoting well-being at all ages, efforts on the health of the mother during pregnancy, childbirth, and the postpartum period is the main agenda [1]. Worldwide, in 2017, 295 000 women died during pregnancy, childbirth, and the postnatal period. Of which 94% of maternal deaths occurred in low-resource settings [2]. Moreover, 2.5 million neonates died globally per annum in 2017 [3].

To decrease maternal and child mortality, the government of Ethiopia has successfully constructed a number of health posts, health centers, and hospitals [4]. Due to these efforts, the national prevalence of maternal mortality was decreased from 871 to 2000 to 412 per 100,000 live births in 2016. Similarly, the neonatal mortality rate decreased from 97 to 1000 live births in 2000 to 29 per 1000 live births in 2016 [5–8]. Despite this improvement, still, a number of maternal and child deaths occurred per year, and seems difficult to achieve the SDG by 2030. Moreover, in Ethiopia, only 26% mothers delivered at health facility in 2016 [8].

To minimize the death of the mother and their child, postnatal care (PNC), care given to the mother and her newborn baby immediately after the birth and for the first six weeks of life, is the most important service [9]. According to the World Health Organization (WHO), all women and newborns should receive a minimum of three postnatal contacts; following or immediately after birth, between 48 and 72 hours, between 7 and 14 days and at six weeks postpartum [9]. These PNC visits have a tremendous advantage for ensuring appropriate breastfeeding practices, to monitor the overall health status of the newborn, to timely diagnose and intervene birth-related complications, and to plan future family planning options [10–13].

Greater than 60% of global maternal deaths occur during the postnatal period in which 45% of postpartum maternal deaths occur within one day of delivery and 65% occur within one week. Similarly, 75% of all neonatal deaths occur during the first week of life [9, 14]. Since the majority of maternal and newborn deaths occur in the first week (especially in the first two days) of delivery, timely and high-quality PNC is crucial. The earlier the clinical conditions of the mother and her newborn baby are detected, the more effectively and easily managed [12, 13, 15–18].

According to different works of literature; maternal education, maternal age, marital status, birth order, maternal occupation, religion, media exposure, wealth status, place of residence, antenatal care (ANC) visit, place of delivery, size of the baby at birth, distance from the health facility, residence, and region are significant factors associated with delayed first PNC attendance [19–24].

Even though delayed PNC visits have a great impact on the survival of the mother and her newborn, still it receives limited attention than other maternal health services, and hence most mothers do not receive PNC services timely [8, 21–23]. Despite this, to the best of our knowledge, limited studies are conducted in Africa including Ethiopia on this issue. Besides, studies conducted elsewhere do not consider the community level factors (they consider factors only at the individual level). Hence, we aimed to determine both the individual and community level factors affecting early postnatal care attendance in Ethiopia. Identifying community level factors is important to take intervention not only at the individual level but also at the community level and we hope this will decrease the problem in a better way. The findings of this study might be used for policymakers, researchers, and health professionals for the betterment of health services after delivery to have good maternal and child health.

## Methods

### Data source, population and sampling technique

This study was based on the recent Ethiopian Demographic and Health Survey (EDHS 2016) data. For this survey, a complete list of 84,915 enumeration areas (EAs) from the Ethiopia Population and Housing Census (PHC) was used as a sampling frame.

The sample for EDHS 2016 has been stratified and chosen in 2 stages. In the first stage 645 EAs were selected (202 in urban areas). In the second level, the newly created household listing selected 28 households per cluster. All necessary information about the sampling strategy, questioner, or other important information exists in the 2016 EDHS report [8]. This data was publicly available. Therefore, we accessed data from the measuredhs.com website after providing a reasonable justification. Finally, for this study, a total weighted sample of 4308 women with a live birth in the two years preceding the 2016 EDHS survey was included.

### Variables of the study

The outcome variable for this study was delayed first PNC attendance, which is defined as the first PNC visit after 2 days (48 hours) of giving birth [8, 23]. The independent variables for this study were further classified as individual level and community level variables. The individual level variables were; maternal education, maternal age, marital status, religion, maternal occupation, household wealth status, birth order, size of the baby at birth, ANC visit, place of delivery, delivery by cesarean section (CS), health insurance coverage, media exposure, and distance from the health facility. Residence, region, community level of media exposure, and community level of women education were the community level variables for this study. These community level variables (community level of media exposure and community levels of women education) were created by aggregating individual-level factors since they were not directly present or recorded in the survey.

#### Operational definitions

Household wealth status: It was created using principal components analysis and it is directly available in the EDHS dataset with the five categories (poorest, poorer, middle rich, and richest [25]. It was re-coded as poor (includes the poorest and poorer category), middle, and rich (includes the rich and richest categories) for our analysis.

Size of the child at birth: it was based on the mother’s perception about the size of the child at birth and was recorded as very small, smaller than average, average, larger than average, and very large in the EDHS data set. We were re-categorized into three categories as small size baby, average size baby, and large size baby small.

Media exposure: Created by combining whether a respondent reads the newspaper, listen to the radio, and watch television and coded as “yes” if the subject was exposed to at least one of the three medias and “no” otherwise.

Community level of media exposure: Was measured by the proportion of women who had exposed to at least one medias; television, radio, or newspaper and categorized based on national median value as low (< 50% of women exposed) and high (≥ 50% of women exposed).

Community level of women education: Measured by the proportion of women with a minimum of primary level of education derived from data on respondents’ level of education. Then, it was categorized using national median value to values: low (< 50% of women have at least primary education) and high (≥ 50% of women have at least primary education).

### Data processing and analysis

To make it suitable for descriptive and analytical analysis, the data were further coded using Stata 14 software. Sample weighting was done to adjust for non-proportional allocation of the sample to strata during the survey process as well as to restore the representativeness of the sample. Since the EDHS data is hierarchical, multilevel logistic regression analysis was fitted. Accordingly, variables with p-value < 0.20 in the bivariable analysis were entered into multivariable analysis, and finally variables with p value < 0.05 were considered as significant factors associated with delayed first PNC attendance.

The measures of community variation (random-effects) were estimated using the intraclass correlation coefficient (ICC), median odds ratio (MOR), and proportional change in variance (PCV) [26–30]. We fitted four models; null model (a model without explanatory variables), model 1(a model containing individual-level factors only), model 2 (a model fitted using community-level factors only), and final or model 3 (a model which examined the effects of both individual and community-level factors). To select the best-fitted model, deviance was used and the model with the lowest deviance (model 3) was selected.

## Results

### Sociodemographic characteristics of respondents

Total weighted samples of 4308 women were used for final analysis. The median age of participants was 27 (IQR = 23–32) years. The majority, 60.49% of participants had no formal education. Around 45.36% of respondents were from poor households. More than half (53.10%) of women had less than four ANC visits during their last pregnancy and 63.78% of study participants gave birth at home. Regarding media exposure and distance from the health facility, 65.44% and 60.50% of study participants had no media exposure and perceived distance from the health facility as a big problem, respectively. The majority (87.93%) of respondents were from rural Ethiopia. Looking at region, 44.44% and 20.34% of participants were from Oromia and Southern Nation Nationalities and People’s region (SNNPR) regions, respectively (Table 1).

**Table 1:** Sociodemographic characteristics of respondents

Variable	Weighted frequency	Weighted percentage
Maternal age(years) 15–19 20–34 35–49	282 3167 859	6.53 73.52 19.94
Maternal education No formal education Primary education Secondary & above	2606 1319 383	60.49 30.61 8.90
Marital status Married Not married	4050 258	94.00 6.00
Religion Orthodox Muslim Protestant Others^a^	1471 1800 882 155	34.13 41.78 20.48 3.60
Maternal occupation Working Not working	1798 2510	41.73 58.27
Household wealth status Poor Middle Rich	1954 890 1464	45.36 20.67 33.98
Size of the child at birth Small Average Large	1234 1790 1284	28.65 41.55 29.80
Birth order One Two & three Four & above	878 1317 2113	20.39 30.57 49.05
Antenatal care visit Less than four Greater or equal to four	2288 2020	53.10 46.90
Place of delivery Home Health facility	2748 1560	63.78 36.22
Covered by health insurance Yes No	164 4144	96.20 3.80
Media exposure No Yes	2819 1489	65.44 34.54
Perceived distance from the health facility Big problem Not a big problem	2606 1702	60.50 39.50
Residence Urban Rural	520 3788	12.07 87.93
Region Tigray Afar Amhara Oromia Somali Benishangul SNNPR Gambela Harari Addis Ababa Dire Dawa	314 43 189 1915 178 45 876 10 10 110 18	7.29 0.99 18.31 44.44 4.13 1.05 20.34 0.24 0.23 2.55 0.43
Community-level media exposure Low High	1959 2348	45.48 54.52
Community-level women education Low High	2011 2297	46.68 53.32

Note; ^a^=catholic, traditional and other religions, *SNNPR* Southern Nation Nationalities and People’s Region

### Random effect and model comparison

Table 2 revealed the random effect result of delayed first PNC attendance. The ICC value in the null model revealed that 39.7% of the total variation in delayed first PNC attendance was due to the difference between clusters. Besides, the MOR in the null model, which was 4.05, indicates that there was a significant variation between clusters. This means when randomly select an individual from two different clusters (one cluster with a higher risk and the other cluster at lower risk), individuals at the cluster with a higher risk of delayed first PNC attendance had 4.05 times higher odds of having a delayed first PNC attendance as compared with their counterparts. As showed by PCV, in the final model (model 3), 96.8% of the variation in delayed first PNC attendance was explained by the final model, a model that incorporates both the individual and community level factors. All three parameters revealed that the effect of clustering was significant. Regarding model fitness, the best fit model was the final model (had the lowest deviance) (Table 2).

**Table 2:** Random effect (measures of variation) and model fitness

Measure of variation	Null model		Model 1	Model 2	Model 3
Community level variance (SE)	2.166(0.276)		0.153(0.089)	0.451(0.113)	0.070(0.079)
ICC	0.397		0.044	0.121	0.021
MOR	4.05[3.43–4.87]		1.45[1.23–1.93]	1.89[1.65–2.26]	1.15[1.09–2.07]
PCV	Reference		0.929	0.792	0.968
Model fitness					
Deviance		3817.4	2732.92	3451.14	2687.96

Note; *ICC* Intra-class Correlation Coefficient, *MOR *Median Odds Ratio, *PCV *Proportional Change in Variance

### Individual and community level factors associated with delayed first PNC attendance

In the bivariable multilevel regression analysis all variables, except marital status and delivery by cesarean section, were significantly associated with delayed first PNC attendance (*p* < 0.2). In multivariable multilevel analysis ANC visit, place of delivery, distance from the health facility, and region were significantly associated with delayed postnatal care attendance (*p* < 0.05). The odds of delayed first PNC attendance was 27% [AOR = 0.73; 95%CI: 0.59, 0.92] lower among mothers who had four or more ANC visit than their counterparts. In addition, the odds of delayed first PNC attendance was 96% [AOR = 0.04; 95%CI: 0.03, 0.05] lower among mothers who gave birth at the health facility than mothers who gave birth at home. Mothers who did not perceive distance from the health facility as a big problem had 27% [AOR = 0.73; 95%CI: 0.58, 0.91] lower odds of delayed first PNC attendance as compared to their counterparts. Moreover, mothers from Oromia and Gambela region had 2.30 [AOR = 2.30; 95%CI: 1.38, 3.83], and 2.01 [AOR = 2.01; 95%CI: 1.13, 3.56] times higher odds of delayed first PNC attendance as compared to mothers from Addis Ababa (Table 3).

**Table 3:** Bi variable and multivariable multilevel analysis of factors associated with delayed first postnatal care attendance in Ethiopia

Variables	Delayed first PNC visit		Odds ratio	
	**No** **N (%)**	**Yes** **N (%)**	**COR (95% CI)**	**AOR (95% CI)**
Maternal age(years) 15–19 20–34 35–49	43(15.21) 550(17.36) 123(14.29)	239(84.79) 2617(82.64) 736(85.71)	1.00 1.21(0.85–1.74) 1.51(1.01–2.27)	1.00 1.14(0.77–1.69) 1.14(0.70–1.88)
Maternal education No formal education Primary education Secondary & above	275(10.56) 277(20.97) 164(42.72)	2331(89.44) 1042(79.03) 219(57.28)	1.00 0.42(0.34–0.52) 0.21(0.16–0.27)	1.00 0.79(0.61–1.03) 0.73(0.53–1.02)
Religion Orthodox Muslim Protestant Others	370(25.14) 204(15.58) 137(11.34) 4(2.71)	1101(74.86) 1596(84.42) 745(88.66) 151(97.29)	1.00 2.30(1.62–3.26) 2.48(1.88–3.28) 7.40(2.64–20.78)	1.00 1.02(0.70–1.50) 1.04(0.77–1.41) 2.49(0.86–7.21)
Maternal occupation Not Working working	370(14.74) 346(19.22)	2140(85.26) 1452(80.78)	1.00 0.76(0.62–0.92)	1.00 0.82(0.67–1.01)
Household wealth status Poor Middle Rich	179(9.15) 128(14.35) 409(29.94)	1775(90.85) 762(85.65) 1055(72.06)	1.00 0.50(0.37–0.67) 0.21(0.16–0.26)	1.00 0.73(0.53–1.01) 0.88(0.65–1.18)
Size of the child at birth Small Average Large	184(14.94) 304(17.00) 227(17.67)	1050(85.06) 1486(83.00) 1057(82.33)	1.00 0.80(0.63–1.01) 0.70(0.54–0.90)	1.00 0.98(0.77–1.25) 0.95(0.73–1.24)
Birth order One Two & three Four & above	183(20.83) 261(19.80) 272(26.86)	695(79.17) 1056(80.20) 1841(87.14)	1.00 1.35(1.07–1.71) 2.05(1.62–2.60)	1.00 0.82(0.63–1.06) 0.73(0.54–1.01)
Covered by Health insurance Yes No	50(30.54) 665(16.06)	114(69.46) 3479(83.94)	0.47(0.29–0.76) 1.00	0.83(0.53–1.31) 1.00
ANC visit <4 ≥4	208(9.06) 508(25.15)	2080(90.94) 1512(74.85)	1.00 0.32(0.26–0.40)	1.00 0.73(0.59–0.92) **
Place of delivery Home Health facility	53(1.93) 663(42.47)	2695(98.07) 897(57.53)	1.00 0.02(0.02–0.03)	1.00 0.04(0.03–0.05) *
Media exposure No Yes	312(11.06) 404(27.12)	2507(88.94) 1085(72.88)	1.00 0.33(0.27–0.40)	1.00 0.89(0.69–1.15)
Perceived distance from the health facility Big problem Not a big problem	264(10.14) 451(26.51)	2342(89.86) 1251(79.49)	1.00 0.35(0.29–0.43)	1.00 0.73(0.58–0.91) **
Residence Urban Rural	235(45.27) 480(12.68)	285(54.73) 3308(87.32)	1.00 6.78(5.15–9.16)	1.00 0.94(0.68–1.30)
Region Addis Ababa Tigray Afar Amhara Oromia Somali Benishangul SNNPR Gambela Harari Dire Dawa	61(55.87) 142(45.36) 5(11.60) 148(18.79) 172(9.00) 21(11.88) 7(14.53) 148(16.88) 2(16.86) 4(37.37) 5(27.77)	49(44.13) 172(54.64) 38(88.40) 642(8121) 1142(91.00) 157(88.22) 38(85.47) 728(83.12) 8(83.14) 6(62.63) 13(72.23)	1.00 1.93(1.13–3.28) 19.47(10.20-37.18) 8.00(4.51–14.19) 17.91(10.08–31.82) 17.98(9.92–32.60) 9.69(5.23–17.98) 8.10(4.66–14.06) 11.14(5.84–21.25) 1.91(1.043–3.51) 3.90(2.09–7.29)	1.00 0.77(0.50–1.20) 1.10(0.61–1.20) 1.35(0.82–2.21) 2.30(1.38–3.83) *** 1.38(0.81–2.38) 1.43(0.83–2.45) 1.44(0.87–2.38) 2.01(1.13–3.56) *** 0.66(0.40–1.10) 1.36(0.82–2.25)
Community level media exposure Low High	177(9.06) 538(22.91)	1782(90.94) 1810(77.09)	1.00 0.17(0.13–0.23)	1.00 0.84(0.64–1.11)
Community level women education Low High	192(9.52) 524(22.81)	1819(90.48) 1773(77.19)	1.00 0.20(0.15–0.27)	1.00 1.18(0.89–1.57)

Note; *AOR* Adjusted Odds Ratio, *COR *Crude Odds Ratio, *CI* Confidence Interval, *PNC* Postnatal Care, *=*p* value ≤ 0.001; **= *p* value less < 0.01; ***=*p* value < 0.05

## Discussion

Maternal and neonatal mortality immediately following birth is devastating and major public health problem especially in countries with a poor clinical setting like Ethiopia. Early intervention of complications that happen during the postpartum period have a paramount advantage and it is one of the main agenda to achieve the SDG 3 [1]. Therefore, this study aimed to determine the individual and community level factors that are associated with delayed first postnatal care attendance in Ethiopia. Identifying these factors is important to take interventions for the problem and minimize maternal and neonatal deaths in the country.

In this study, both individual level (ANC visit, place of delivery and distance from the health facility) and community level factors (region) were significantly associated with delayed first postnatal care attendance in Ethiopia. Consistent with studies done in Ethiopia [21] and South Sudan [24], in this study women who perceive distance from the health facility as a big problem had higher odds of delayed PNC visits. This is due to mothers who are far from the nearest health facility are less likely to receive appropriate maternal health services such as ANC visits and delivery at the health facility which have their impact on early initiation of PNC visits. Besides, issues related to distance from the health facilities such as lack of transport and its costs, and lack of suitable roads might be a reason for delayed maternal health service utilization early PNC visits [31, 32].

Antenatal care is a significant predictor of delayed PNC attendance in this study. Those women who had four or more ANC visits had lower odds of delayed PNC attendance and this is congruent with studies done in Zambia and Uganda [19, 23]. This might be because mothers who are fully attended ANC might receive appropriate and adequate counseling about danger signs and complications during the postnatal period and the benefits of taking the PNC early [19, 33–35].

In this study, place of delivery was another significantly associated factor with delayed PNC attendance. Women who gave birth in the health facility had lower odds of delayed PNC attendance as compared to women who gave birth at home. This finding is in line with cross-sectional studies done in Ethiopia [24] and Uganda [19]. This might be because women delivered in the health facility had a great opportunity for health education and information related to the services given during the post-natal period and the advantages of early PNC visits for the health of the mother and her newborn [20, 36].

Furthermore, region was another important factor that was associated with delayed PNC attendance in which women from Oromia and Gambela region had higher odds of delayed first PNC attendance. Other studies also revealed region as an important factor for the utilization of maternal health services including postnatal care [24, 37, 38]. The possible explanation for this might be due to the sociocultural difference between regions as well as the differences in the accessibility and quality of care.

This study has both strengths and limitations. The key strength is its representativeness as it was based on nationally representative data. In addition, it was based on an appropriate statistical method (multilevel analysis) to complement the data’s hierarchical nature. Therefore, the study findings could help policymakers to make wise decisions, and it could be used as a baseline for future researchers/studies. As a limitation, due to the retrospective nature in the assessment of the timing of the PNC visit, there might be a possibility of recall bias. Besides, due to the cross-sectional nature of the data, causality cannot be established between the dependent and independent variables. Moreover, because it is based on the information contained in the data set, certain variables/confounders such as complications during birth and the availability of quality health care are missed in the analysis.

## Conclusions

In this study, both individual and community level factors were associated with delayed first PNC attendance. Among individual-level factors; having four or more antenatal care visits, not perceiving distance from the health facility as a big problem, and delivery at the health facility were associated with lower odds of delayed first PNC attendance. Of community level factors, region (being in Oromia and Gambela regions) was associated with higher odds of delayed PNC attendance. Therefore, it is better if health professionals, possible stakeholders, and policymakers give special attention on antenatal care visit, institutional delivery, and appropriate distributions of maternal health services in different regions especially in areas that are far apart from the health facilities to minimize delayed first PNC attendance.

## Data Availability

All result-based data are within the manuscript and the data set can be accessed online from www.measuredhs.com/data.

## Abbreviations

ANC: Antenatal Care
AOR: Adjusted Odds ratio
EAs: Enumeration Areas
EDHS: Ethiopian Demographic and Health Survey
ICC: Intraclass Correlation Coefficient
MOR: Median odds ratio
PCV: Proportional Change in Variance
PHC: Ethiopia Population and Housing Census
PNC: Postnatal Care
SDG3: Sustainable Development Goal three
SNNPR: Southern Nation Nationalities and People’s Region
WHO: World Health Organization
